# Prognostic significance of pretreatment PET parameters in inoperable, node-positive NSCLC patients with poor prognostic factors undergoing hypofractionated radiotherapy: a single-institution retrospective study

**DOI:** 10.1186/s41824-024-00220-w

**Published:** 2024-10-08

**Authors:** Annemarie Barbara Zinn, Saskia Kenndoff, Adrien Holzgreve, Lukas Käsmann, Julian Elias Guggenberger, Svenja Hering, Sina Mansoorian, Nina-Sophie Schmidt-Hegemann, Niels Reinmuth, Amanda Tufman, Julien Dinkel, Farkhad Manapov, Claus Belka, Chukwuka Eze

**Affiliations:** 1 Department of Radiation Oncology, University Hospital, LMU Munich, Munich, Germany; 2 Department of Nuclear Medicine, University Hospital, LMU Munich, Marchioninistr, 15, 81377 Munich, Germany; 3 German Cancer Consortium (DKTK), partner site Munich; and German Cancer Research Center (DKFZ), Heidelberg, Germany; 4 Comprehensive Pneumology Center Munich (CPC-M), Member of the German Center for Lung Research (DZL), Munich, Germany; 5 Department of Oncology, Asklepios Lung Clinic Munich-Gauting, Gauting, Germany; 6 Department of Medicine V, University Hospital, Munich, Germany; 7 Department of Radiology, University Hospital, Munich, Germany; 8 Department of Radiology, Asklepios Lung Clinic Munich-Gauting, Gauting, Germany; 9 Bavarian Cancer Research Center (BZKF), Munich, Germany

**Keywords:** Baseline PET, Hypofractionation, NSCLC, Poor prognostic factors, tMTV

## Abstract

**Background:**

Node-positive non-small cell lung cancers (NSCLCs) present a challenge for treatment decisions, particularly in patients ineligible for concurrent chemoradiotherapy (CRT) due to poor performance status and compromised lung function. We aimed to investigate the prognostic value of pretreatment positron emission tomography (PET) parameters in high-risk patients undergoing hypofractionated radiotherapy.

**Methods:**

A retrospective analysis was conducted on 42 consecutive patients with inoperable node-positive NSCLC, who underwent hypofractionated radiotherapy between 2014 and 2021 at a single institution. Clinical, treatment-related, and [^18^F]FDG PET-based parameters were correlated with progression-free survival (PFS) and overall survival (OS). Median dichotomisation was performed to establish risk groups. Statistical analyses included univariable and multivariable Cox regression and Kaplan-Meier survival analyses.

**Results:**

After a median follow-up of 47.1 months (range: 0.5-101.7), the median PFS and OS were 11.5 months (95% CI: 7.4-22.0), and 24.3 months (95% CI: 14.1-31.8). In univariable Cox regression analysis, significant predictors of PFS included receipt of salvage systemic treatment (p=0.007), SUVmax (p=0.032), and tMTV (p=0.038). Similarly, ECOG-PS (p=0.014), Histology (p=0.046), and tMTV (p=0.028) were significant predictors of OS. Multivariable Cox regression analysis (MVA) identified SUVmax as a significant predictor for PFS [HR: 2.29 (95% CI: 1.02-5.15); p=0.044]. For OS, ECOG-PS remained a significant prognosticator [HR: 3.53 (95% CI: 1.49-8.39); p=0.004], and tMTV approached significance [HR: 2.24 (95% CI: 0.95-5.26); p=0.065]. Furthermore, the high tMTV group exhibited a median PFS of 5.3 months [95% CI: 2.8-10.4], while the low tMTV group had a PFS of 15.2 months [95% CI: 10.1-33.5] (p=0.038, log-rank test). Median OS was 33.5 months [95% CI: 18.3-56.8] for tMTV ≤ 36.6 ml vs. 14.1 months [95% CI: 8.1-27.2] for tMTV > 36.6 ml (p=0.028, log-rank test).

**Conclusion:**

Pretreatment PET parameters, especially tMTV, hold promise as prognostic indicators in NSCLC patients undergoing hypofractionated radiotherapy. The study highlights the potential of PET metrics as biomarkers for patient stratification.

**Supplementary Information:**

The online version contains supplementary material available at 10.1186/s41824-024-00220-w.

## Introduction

Node-positive non-small cell lung cancers (NSCLCs) represent a heterogeneous group of lung tumours (Daly et al. 2022). Recommendations within interdisciplinary tumour boards may be influenced by risk factors, including comorbidities and impaired lung function, potentially leading to the recommendation of radiotherapy (XRT) as a stand-alone intervention with recent studies demonstrating a rationale for combining XRT and immunotherapy (abbreviated iRT henceforth) in this context (Yamada et al. 2023; Filippi et al. 2023; Bozorgmehr et al. 2023).

When radiotherapy is administered without concurrent chemotherapy, one potential strategy to reduce overall treatment time and mitigate tumour repopulation is the use of hypofractionation, following current recommendations from the American Society of Clinical Oncology (ASCO) (Daly et al. 2022). However, a recent phase 3 randomised study failed to demonstrate the superiority of hypofractionation in patients with poor performance status ineligible for concurrent chemoradiation (CRT) (Iyengar et al. 2021). Notably, various guidelines and literature reviews endorse the use of [^18^F]Fluorodeoxyglucose positron emission tomography/computed tomography ([^18^F]FDG PET/CT) for staging and treatment planning in NSCLC (Nestle et al. 2006; Vaz et al. 2022; Manapov et al. 2022; Unterrainer et al. 2020).

The prognostic significance of pretreatment PET parameters within the context of diverse therapeutic strategies is actively under investigation (Eze et al. 2021a). The most examined metrics in this context include SUVmax (Maximum Standardised Uptake Value), MTV (Metabolic Tumour Volume), and TLG (Total Lesion Glycolysis). While SUVmax is already employed in clinical routine and indicates the highest FDG uptake in a single voxel within a region of interest (ROI), both MTV and TLG are volumetric parameters providing information about both the tumour size and its metabolic activity. Mathematically, TLG is the product of MTV and the corresponding value of the mean SUV (SUVmean) (Pellegrino et al. 2021).

Given the expanding literature on iRT in this setting, our objective was to explore the correlation between baseline PET parameters and outcomes following radiotherapy in inoperable, node-positive NSCLC patients. These patients, due to compromised lung function and additional poor risk factors, were ineligible for definitive concurrent CRT. Previously, our group published clinical outcomes for this distinct cohort of patients treated at our institution (Eze et al. 2019, b, 2022).

## Methods

### Patient characteristics

Between 2014 and 2021, consecutive patients were retrospectively and prospectively enrolled in a single-institution observational cohort study approved by the Ludwig Maximilian University of Munich institutional review board (reference numbers 17–230 and 17–233). Our study included patients meeting specific criteria: cytologically/histologically confirmed NSCLC, Eastern Cooperative Oncology Group (ECOG) performance status (PS) ≥ 1, inoperable node-positive clinical stage IIB (N1)/ III (TNM 8th edition (Detterbeck et al. 2017), or recurrent disease ineligible for concurrent CRT. All patients had a forced expiratory volume in 1 second (FEV1) ≤ 1.0 L and/or single-breath diffusing capacity of the lung for carbon monoxide (DLCO-SB) ≤ 40% and/or were on long-term oxygen therapy (LTOT). Additionally, included in this analysis were all patients who underwent [^18^F]FDG PET/CT scans before the initiation of definitive therapy: 42/47 (89%) patients from our previous analysis (Eze et al. 2022). All patients received 3D conformal radiotherapy (3DCRT) or intensity-modulated radiotherapy (IMRT) administered in 13 to 16 daily fractions, five days a week. The total dose ranged from 42.0 to 49.0 Gy (2.8–3.5 Gy per fraction), resulting in biologically equivalent doses in 2-Gy fractions (EQD2) and biologically effective dose (BED), assuming an α/β ratio of 10 for NSCLC of 45.5–55.1 Gy and 54.6–66.2 Gy, respectively. There was no difference in the radiation dose for the primary tumour and the lymph nodes.

### Image acquisition and data evaluation

Image acquisition has been previously described. To reiterate, the acquisition of [^18^F]FDG PET/CT images followed recommended guidelines from the European Association of Nuclear Medicine (EANM), the Society of Nuclear Medicine and Molecular Imaging (SNMMI), and the European Society for Radiotherapy and Oncology (ESTRO) (Vaz et al. 2022; Unterrainer et al. 2022; Holzgreve et al. 2023). Imaging began approximately 60 min after the administration of [^18^F]FDG, with additional administration of 10–20 mg furosemide and 10–20 mg butylscopolamine whenever possible. PET imaging reconstruction was performed as described previously (Unterrainer et al. 2022). SUV quantification was based on total body weight. PET imaging was simultaneously acquired with a diagnostic CT scan after the administration of 350 mg of iomeprol (Imeron^®^) at 1.5 ml/kg body weight (portal-venous phase).

Two experienced nuclear medicine physicians/radiologists assessed the [^18^F]FDG PET/CT images. They measured the maximum standardised uptake value (SUVmax) of tumoural lesions. Mean activity of the spleen (SUVspleen) and the bone marrow (SUVBM) were derived using a 3.0 cm and a 1.5 cm spherical volume of interest (VOI) placed in the center of the spleen or lumbar vertebrae, respectively. If no relevant (degenerative) changes were present, bone marrow uptake was determined using the mean uptake of L4 and L5. Alternatively, other lumbar vertebrae were used. (Holzgreve et al. 2023). Short and long-axis diameters of tumour manifestations were measured on CT images. For spleen volume estimation, a three-dimensional approach using the product of the spleen length, maximal width, and thickness on CT images was employed (Cools et al. 1983).

### PET parameters

In this study, we examined volumetric and intensity-based PET metrics separately for the primary tumour and the lymph nodes. For volumetric parameters, we considered MTV and TLG, whereas, for intensity-based metrics, we analysed SUVmax, SUVmean, and SUVpeak. For each parameter a risk-based classification was performed using median dichotomisation.

### Clinical and treatment-related parameters

Beyond PET-based parameters, clinical parameters were assessed, including age, sex, T-descriptor, N-descriptor, UICC stage, performance status, Charlson comorbidity index (CCI) with oncological diagnosis, histological subtypes, administration of induction systemic treatment, and administration of salvage systemic treatment.

### Outcome parameters

Clinical and image-derived parameters were correlated with patients’ progression-free survival (PFS) and overall survival (OS). PFS was defined as the duration from the last day of XRT to either locoregional or systemic progression or the patient’s death. OS was defined as the duration from the end of XRT until the patient’s death from any cause or their last recorded follow-up.

### Statistics

The median follow-up duration was calculated using the reverse Kaplan-Meier method from the final day of XRT until the last follow-up or the point at which follow-up was lost. Descriptive statistics were presented as mean (standard deviation: SD) or median (range). Kaplan-Meier curves, log-rank tests, and median survival with a 95% confidence interval (CI) were employed for the univariable comparison of PFS and OS. Due to the small sample size, variables with (borderline) significance in the univariable analysis (*p* < 0.1) were included in the multivariable Cox regression analysis. Results were displayed as hazard ratio (HR) and CI. Statistical significance was defined as two-tailed p-values < 0.05, and all analyses were performed using IBM^®^ SPSS^®^ Statistics version 27 and OriginPro, Version 2021b.

## Results

From 2014 to 2021, 42 patients diagnosed with stage IIB (N1)/III or recurrent NSCLC, and experiencing compromised lung function, characterised by LTOT (*n* = 16/38.1%), DLCO-SB ≤ 40% (*n* = 30/71.4%), or FEV1 ≤ 1 L (*n* = 14/33.3%), were treated with hypofractionated radiotherapy at our institution. The multidisciplinary tumour board deemed all 42 patients ineligible for concurrent CRT, leading to their referral for XRT alone. All patients underwent pretreatment [^18^F]FDG PET/CT. Notably, 16 patients received induction systemic therapy, prompting a subgroup analysis (Supplement files). All 42 patients completed radiotherapy.

The median age of the cohort was 72 years (range: 52–88), comprising 24 men (57%) and 18 women (43%). After a median follow-up of 47.1 months (range: 0.5-101.7), the median PFS and OS were 11.5 months (95% CI: 7.4–22.0), and 24.3 months (95% CI: 14.1–31.8), respectively. The risk-based classification was performed using median dichotomisation.

In the univariable Cox regression analysis, significant predictors of PFS included receipt of salvage systemic treatment (*p* = 0.007), SUVmax (*p* = 0.032), and tMTV (*p* = 0.038). Similarly, ECOG-PS (*p* = 0.014), Histology (*p* = 0.046), and tMTV (*p* = 0.028) were significant predictors of OS. Multivariable Cox regression analysis (MVA) identified SUVmax as a significant predictor for PFS [HR: 2.29 (95% CI 1.02–5.15); *p* = 0.044], with salvage systemic therapy bordering significance [HR: 2.36 (95% CI0.95-5.83); *p* = 0.064]. For OS, ECOG-PS remained a significant prognosticator [HR: 3.53 (95% CI 1.49–8.39); *p* = 0.004], and tMTV approached significance [HR: 2.24 (95% CI 0.95–5.26); *p* = 0.065] (Table 1).

**Fig. 1:**
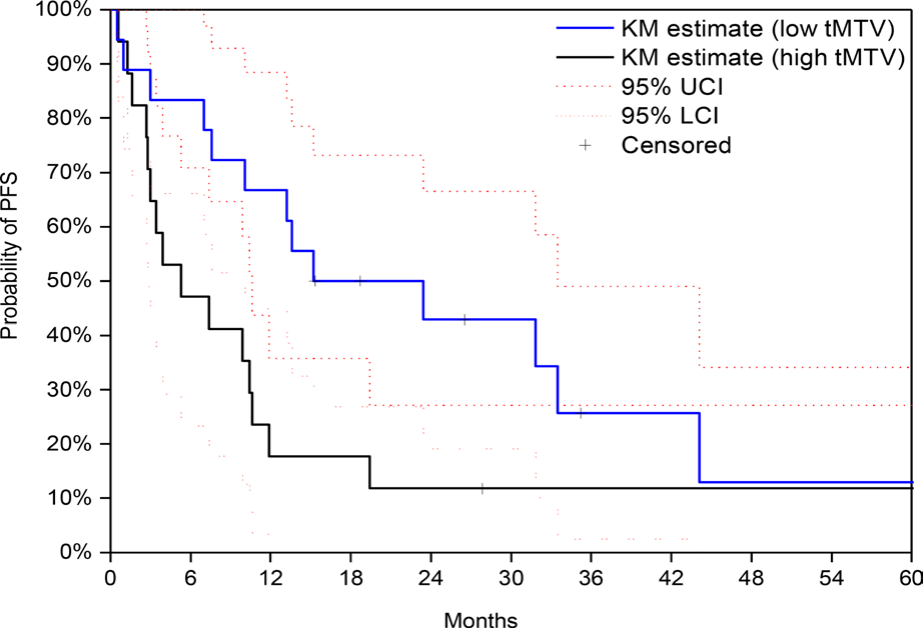
Kaplan-Meier estimate of PFS of low vs. high tMTV. The high-risk group exhibited a median PFS of 5.3 months [95% CI: 2.8–10.4], while the low-risk group had a PFS of 15.2 months [95% CI: 10.1–33.5] (*p* = 0.038, log-rank test)

**Fig. 2:**
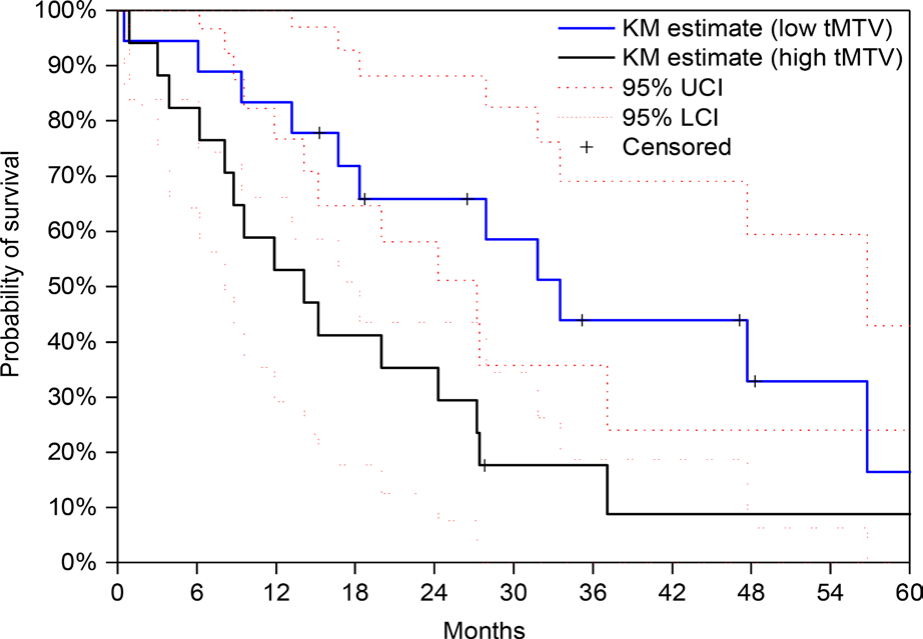
Kaplan-Meier estimate of OS. Median OS was 33.5 months [95% CI: 18.3–56.8] for tMTV ≤ 36.6 ml vs. 14.1 months [95% CI: 8.1–27.2] for tMTV > 36.6 ml (*p* = 0.028, log-rank test)

The high-risk group exhibited a median PFS of 5.3 months [95% CI: 2.8–10.4], while the low-risk group had a PFS of 15.2 months [95% CI: 10.1–33.5] (*p* = 0.038, log-rank test) (Figure 1). Median OS was 33.5 months [95% CI: 18.3–56.8] for tMTV ≤ 36.6 ml vs. 14.1 months [95% CI: 8.1–27.2] for tMTV > 36.6 ml (*p* = 0.028, log-rank test) (Figure 2). Univariable analysis (UVA) revealed a significant association between SUVmax and PFS: 18/35 (51%) patients with SUVmax ≤ 12 had a significantly longer PFS of 13.6 months (95% CI: 10.6–44.1) compared to 5.3 months (95% CI: 2.8–9.9) in the group with SUVmax > 12 (*p* = 0.032).

**Table 1:** Univariable and multivariable analysis of the entire cohort regarding progression-free and overall survival

Entire cohort		Univariable analysis (*p*-value)		Multivariable analysis (*p*-value) [HR (95% CI)]	
	No. of Patients (%)	OS	PFS	OS	PFS
**Age**,** years**					
≥ 70	24 (57)	0.839	0.528		
< 70	18 (43)				
**Sex**					
Male	24 (57)	0.686	0.478		
Female	18 (43)				
**T category**					
Tx- T2	17 (40)	0.185	0.207		
T3- T4	25 (60)				
**N category**					
N1	9 (21.4)	0.13	0.612		
N2	19 (45.2)				
N3	14 (33.3)				
**Stage IIIC/recurrent**					
Yes	20 (48)	0.969	0.655		
No	22 (52)				
**ECOG- PS**					
1	25 (60)	**0.014**	0.206	**0.004**	
2 − 3	17 (40)			[3.53 (1.49–8.39)]	
**CCI**					
4–6	26 (62)	0.132	0.431		
≥7	16 (38)				
**Histology**					
SCC	17 (40)	**0.046**	0.149	0.213	
Non-SCC	25 (60)			[0.57 (0.24–1.38)]	
**Induction systemic therapy**					
Yes	16 (38)	0.583	0.639		
No	26 (62)				
**Salvage systemic therapy**					
Yes	9 (21)	0.281	**0.007**		0.064
No	33 (79)				[2.36 (0.95–5.83)]
**tMTV**					
≤ 36.6 ml	18	**0.028**	**0.038**	0.065	0.291
> 36.6 ml	17			[2.24 (0.95–5.26)]	[1.57 (0.68 − 3.6)]
**SUVmax**					
≤ 12	18	0.215	**0.032**		**0.044**
> 12	17				[2.29 (1.02–5.15)]

Abbreviations CCI: Charlson Comorbidity Index, ECOG-PS: Eastern Cooperative Oncology Group- Performance Status, ml: millilitre, No: number, OS: Overall Survival, PFS: Progression-Free Survival, SCC: Squamous Cell Carcinoma, SUV_max_: Maximum Standardised Uptake Value, tMTV: total Metabolic Tumour Volume

Moreover, in the univariable analysis, none of the other PET metrics (primary or nodal) demonstrated a notable association with progression-free survival (PFS) and overall survival (OS), as indicated in Table 2.

A subgroup analysis for 26/42 (62%) patients who did not receive induction chemotherapy had a median PFS and OS of 11.5 months (95% CI: 7.6–28.7) and 27.4 months (95% CI: 16.7–33.5), respectively. In this subgroup, the median tMTV was 26.2 ml. UVA revealed significant differences in PFS (*p* < 0.001) and OS (*p* = 0.012): higher tMTV was associated with a median PFS of 5.3 months [95% CI: 2.7–10.4] and OS of 15.2 months [95% CI: 8.8–27.2], compared to a median PFS of 31.8 months [95% CI: 15.2-not reached] and OS of 33.5 months [95% CI: 27.9-not reached] in the lower tMTV group. Similar associations were observed between pMTV and PFS (*p* = 0.003) and OS (*p* = 0.061). Additionally, TLG, SUVmax, pSUVmean, pSUVpeak, nSUVmean, and nSUVpeak demonstrated significant associations with PFS and OS (Supplementary Table 2). Multivariable analyses were not conducted due to multicollinearity among these parameters for both PFS and OS.

**Table 2:** Univariable analysis of PET-based metrics in the entire cohort regarding progression-free and overall survival

Entire cohort	Median value	Univariable analysis: (*p*-value)	
		OS	PFS
tMTV (ml)	36.6	**0.028**	**0.038**
pMTV (ml)	34.9	0.338	0.103
nMTV (ml)	10.6	0.453	0.481
SUVmax	12	0.215	**0.032**
pSUVmax	12.5	0.32	0.061
nSUVmax	8.6	0.369	0.42
TLG	211.4	0.338	0.103
pSUVmean	5.5	0.538	0.172
nSUVmean	4.3	0.604	0.548
pSUVpeak	10.8	0.697	0.179
nSUVpeak	8.3	0.979	0.538

Abbreviations MTV: Metabolic Tumour Volume, n: nodal, p: primary, SUVmax: Maximum Standardised Uptake Value, t: total, TLG: Total Lesion Glycolysis

## Discussion

We previously demonstrated the safety and efficacy of moderately hypofractionated radiotherapy as a treatment option for non-metastatic, node-positive NSCLC patients ineligible for surgery or concurrent CRT due to poor performance status and/or severely limited lung function (Eze et al. 2022). This high-risk patient group, characterised by shared adverse prognostic factors and oncological heterogeneity, requires further investigation. PET parameters are proven to predict PFS and OS irrespective of TNM stage and treatment modality (Eze et al. 2021a). The primary focus is on validating pretreatment FDG-PET parameters as prognostic indicators for outcomes and survival in these high-risk patients. Notably, while existing studies have predominantly addressed the role of baseline PET parameters in patients with locally advanced NSCLC undergoing chemoradiation or radiotherapy, there is a notable dearth of data concerning the significance of baseline PET parameters in patients treated exclusively with hypofractionation (Manapov et al. 2022; Eze et al. 2021a).

The key finding in our study is the robust prognostic value of tMTV. In univariable analysis, tMTV emerged as a significant predictor for both OS and PFS across the entire cohort. On MVA, tMTV remained a borderline significant prognosticator for OS. Among common PET parameters, MTV accurately reflects the underlying tumour burden, as suggested by Lee et al. (Lee et al. 2012). Their pilot study revealed a statistical correlation between MTV and OS and PFS across various treatments, including surgery, radiotherapy, or chemotherapy with definitive or palliative intent. A subsequent meta-analysis by Im et al. further confirmed the prognostic impact of MTV, identifying it as an independent marker for OS (Im et al. 2015). This trend holds true for more homogeneous cohorts, such as early-stage NSCLC treated with stereotactic body radiotherapy and non-metastatic node-positive NSCLC treated with CRT (Chin et al. 2018; Dosani et al. 2019; Bazan et al. 2017; Sharma et al. 2018).

Despite consensus on the prognostic value of MTV, specific thresholds remain unclear. Our study, like others, opted for a median split, setting the threshold at a median tMTV of 36.6 ml. This approach aligns with a secondary analysis of the American College of Radiology Imaging Network (ACRIN) 6668/Radiation Therapy Oncology Group 0235 study, where patients with inoperable stage II/III treated with CRT demonstrated significantly worse median overall survival with a pre-MTV > 32 ml (Bazan et al. 2017). Our findings underscore the need for defining thresholds as prognostic biomarkers.

Surprisingly, in the entire cohort, no significant correlations were found between nMTV and PFS or OS, consistent with an Australian study in a comparable patient cohort with inoperable node-positive stage II-III NSCLC after CRT/RT (Alipour et al. 2021). Further, a study by Wang et al. on stage I-III NSCLC patients undergoing CRT/RT also highlighted the primary prognostic significance of tMTV over nMTV or pMTV (Wang et al. 2016).

In the subgroup analysis of patients without induction therapy, pMTV gained prominence, demonstrating a significant association with PFS and a borderline significant correlation with OS in the UVA. A study by Van Diessen et al. on stage IIIA-B NSCLC patients undergoing concurrent CRT revealed the stronger impact of volumetric and intensity PET metrics of the primary tumour compared to lymph nodes (Diessen et al. 2020).

While SUVmax, in the entire cohort, showed a significant association with PFS in the MVA, its prognostic significance remains unclear, as many studies have failed to establish consistent correlations (Im et al. 2015; Alipour et al. 2021; Fontaine et al. 2021; Takahashi et al. 2016). Potential explanations include susceptibility to artifacts, especially with high-resolution modern scanners, or variations in FDG avidity between different histologies (Boellaard et al. 2004; Hicks 2022). Further research is needed to elucidate the role of SUVmax in predicting outcomes in this context.

In our investigation of node-positive NSCLC patients ineligible for concurrent chemoradiotherapy (CRT), we observed encouraging median PFS and OS of 11.5 months and 24.3 months, respectively. These outcomes compare favourably with other relevant studies, underscoring the importance of alternative treatment approaches for this specific patient cohort. Our findings align with the Japanese randomised phase 3 study (JCOG0301) and an Italian study on moderately hypofractionated XRT (Atagi et al. 2012; Valeriani et al. 2019). Notably, patients in our cohort, who were ineligible for concurrent CRT, demonstrated competitive survival rates. This further emphasises the evolving landscape of treatment strategies and the need for tailored approaches for patients with distinct clinical profiles.

The discussion delves into the ongoing exploration of combining durvalumab with conventional or hypofractionated radiotherapy in high-risk NSCLC patients. This strategy, while promising in enhancing treatment efficacy, is not without challenges, as highlighted by studies such as SPIRAL-RT/JMA-IIA00434 and the German TRADE-hypo trial (Yamada et al. 2023; Bozorgmehr et al. 2023). The increased risk of pneumonitis, particularly when durvalumab is administered concomitantly with radiotherapy, necessitates a careful evaluation of the risk-benefit profile.

Preliminary data from the Italian DUART study, presented at ESMO 2023, introduces another dimension to the discussion (Filippi et al. 2023). Administering durvalumab after standard or palliative radiotherapy in this study, with slightly lower equivalent doses in the palliative XRT arm than in our cohort, underscores the adaptability of treatment strategies. However, the observed median PFS and OS rates in the palliative XRT group emphasise the complexity of balancing therapeutic intensification and potential risks.

With an aging population, our study accentuates the growing importance of treatment strategies that do not involve concurrent CRT. The intricate balance between efficacy and safety is highlighted, prompting a reevaluation of conventional treatment paradigms. A pivotal aspect discussed is the role of biomarkers in stratifying patients and identifying those who could benefit from therapy intensification. PET-based metrics emerge as potential candidates for patient classification, with tMTV standing out as a prognostic predictor for both OS and PFS. The independence of tMTV as a survival predictor underscores its potential as a valuable tool for refining treatment decisions.

Acknowledging the limitations of our study, including its retrospective nature, limited sample size, and the inherent heterogeneity of the patient cohort, prompts a cautious interpretation of the results. Variability in PET/CT examinations across different locations further adds a layer of complexity. Future studies should carefully consider these aspects and delve deeper into the intricate interplay of tumour biology in diverse patient populations.

In summary, our study affirms the prognostic value of tMTV in non-metastatic, node-positive NSCLC patients ineligible for surgery or concurrent CRT. While nMTV did not show significant correlations, pMTV demonstrated significance in the subgroup without induction therapy. The role of SUVmax remains unclear and warrants additional investigation. The establishment of standardised thresholds for these PET parameters is crucial for their effective utilisation as prognostic biomarkers in this high-risk patient population.

## Conclusion

In conclusion, our study sheds light on the outcomes of a unique subset of NSCLC patients, offering insights into the challenges and opportunities in treatment paradigms. While tMTV emerges as a promising prognostic indicator, the discussion underscores the need for larger studies to elucidate the prognostic value of SUVmax. The dynamic landscape of radiotherapy and immunotherapy combinations and the potential of biomarkers to guide personalised approaches provide a fertile ground for future research and clinical advancements.

## Electronic supplementary material

Below is the link to the electronic supplementary material.

Supplementary Material 1

## Data Availability

The datasets generated during and/or analysed during the current study are available from the corresponding author on reasonable request.

## Abbreviations

ASCO: American Society of Clinical Oncology
BED: Biologically effective dose
CCI: Charlson Comorbidity Index
CI: Confidence interval
CRT: Chemoradiation
DLCO-SB: Diffusing capacity of the lung for carbon monoxide
EANM: European Association of Nuclear Medicine
ECOG-PS: Eastern Cooperative Oncology Group- Performance Status
ESTRO: European Society for Radiotherapy and Oncology
EQD2: Biologically equivalent doses in 2-Gy fractions
FEV1: Forced expiratory volume in 1 s
HR: Hazard ratio
IMRT: Intensity-modulated radiotherapy
iRT: Immunoradiotherapy
LTOT: Long-term oxygen therapy
MTV: Metabolic Tumour Volume
MVA: Multivariable analysis
n: Nodal
NSCLC: Non-small cell lung cancer
OS: Overall Survival
p: Primary
PFS: Progression-Free Survival
ROI: Region of interest
SCC: Squamous Cell Carcinoma
SD: Standard deviation
SNMMI: Society of Nuclear Medicine and Molecular Imaging
SUV: Standardised Uptake Value
SUVmax: Maximum SUV
SUVmean: Mean SUV
SUVBM: SUVmean of the bone marrow
SUVspleen: SUVmean of the spleen
t: Total
TLG: Total Lesion Glycolysis
UVA: Univariable analysis
VOI: Volume of interest
XRT: Radiotherapy
3DCRT: 3D conformal radiotherapy
[^18^F]FDG PET/CT: [^18^F]Fluorodeoxyglucose positron emission tomography/computed tomography
