# Supplementary material for: Prognostic significance of pretreatment PET parameters in inoperable, node-positive NSCLC patients with poor prognostic factors undergoing hypofractionated radiotherapy: a single-institution retrospective study
Source: EJNMMI Rep. 2024 Oct 8;8(1):32. doi: 10.1186/s41824-024-00220-w (PMC11458843; doi:10.1186/s41824-024-00220-w)
Supplement: Supplementary file 1 — Supplementary Material 1 [file 41824_2024_220_MOESM1_ESM.docx]

Supplementary Table 1: Univariable analysis of the patient subgroup without induction therapy regarding progression-free and overall survival

| Patient subgroup without Induction  therapy |  | Univariable analysis  (*p*-value) | |
| --- | --- | --- | --- |
|  | **No. of Patients**  **(%)** | **OS** | **PFS** |
| Age, y  ≥70 <70 | 18 (69)  8 (31) | .833 | .928 |
| Sex  Male  Female | 15 (58) 11 (42) | .528 | .498 |
| T category  Tx- T2  T3- T4 | 13 (50) 13 (50) | .287 | .368 |
| N category  N1 N2 N3 | 6 (23)  13 (50)  7 (27) | **.034** | .535 |
| Stage IIIC/recurrent  Yes No | 12 (46)  14 (54) | .852 | .349 |
| ECOG- PS  1 2- 3 | 15 (58)  11 (42) | .112 | .753 |
| CCI  4- 6  ≥7 | 12 (46)  14 (54) | .153 | .472 |
| Histology  SCC  Non-SCC | 13 (50)  13 (50) | **.028** | .367 |
| Salvage systemic therapy  Yes No | 5 (19)  21 (81) | .515 | .148 |

Supplementary Table 2: Univariable analysis of primary/nodal PET metrics in the patient subgroup without induction therapy regarding progression-free and overall survival

| Subgroup without Induction therapy | Median  value | Univariable Analysis:  (*p*-value) | |
| --- | --- | --- | --- |
|  |  | OS | PFS |
| tMTV (ml) | 26.2 | **.012** | **<.001** |
| pMTV (ml) | 28 | .061 | **.003** |
| nMTV (ml) | 10.6 | .492 | .398 |
| SUVmax | 12 | .096 | **.009** |
| pSUVmax | 13 | .527 | .269 |
| nSUVmax | 8.2 | .071 | .155 |
| TLG | 175.4 | .091 | **<.001** |
| pSUVmean | 5.3 | .205 | **.027** |
| nSUVmean | 4 | **.048** | .068 |
| pSUVpeak | 11 | .184 | **.031** |
| nSUVpeak | 7.6 | **.022** | .095 |

Supplementary Figure 1: Kaplan-Meier estimate of the patient subgroup without induction therapy regarding progression-free survival: high tMTV was associated with a median PFS of 5.3 months [95% CI: 2.7-10.4] vs. 31.8 months [95% CI: 15.2-not reached] in the low tMTV subgroup.

**

Supplementary Figure 2: Kaplan-Meier estimate of the patient subgroup without induction therapy regarding overall survival: high tMTV was associated with a median OS of 15.2 months [95% CI: 8.8-27.2] vs. 33.5 months [95% CI: 27.9-not reached] in the low tMTV group.
